# Architecting a partial thickness cartilage substitute with mimetic, self-assembling hydrogels†

**DOI:** 10.1039/d5tb00050e

**Published:** 2025-04-11

**Authors:** Olivia F. Dingus, Kathleen A. Parrish, Andrew P. Haney, Cesar A. Ramirez, Melissa A. Grunlan

**Affiliations:** a Department of Biomedical Engineering, Texas A&M University College Station TX 77843-3003 USA mgrunlan@tamu.edu; b Department of Materials Science & Engineering, Texas A&M University College Station TX 77843-3003 USA; c Department of Chemistry, Texas A&M University College Station TX 77843-3003 USA

## Abstract

Restoration of partial thickness chondral defects (PTCDs) may be achieved with a synthetic substitute that mimics the discrete mechanical properties of the superficial and transitional chondral layers. Moreover, innate adhesivity of the two components would enable the facile construction and integrity of this bilayered system. Herein, we report a PTCD bilayered substitute formed by triple network (TN) hydrogels that leverage electrostatic charge interactions to achieve mechanical mimicry and self-assembly. TN hydrogels were formed with a polyampholyte 3rd network of five different charge composition (*i.e.*, ratio of cationic and anionic monomers), as well as two crosslink densities. All TN hydrogels exhibited cartilage-like hydration. A single superficial-like chondral layer TN hydrogel, with a somewhat more anionic 3rd network, was identified having mimetic compressive modulus (∼1.8 MPa) and strength (∼13 MPa). Additionally, three transitional-like chondral layer candidates were identified, including two TN hydrogels with a more cationic 3rd network in addition to the TN hydrogel with a ‘cationic-only’ 3rd network. The adhesivity of the superficial layer and the three transitional layer candidates was found to be robust (∼>100 kPa), wherein the bilayered construct exhibited cohesive rather than adhesive failure.

## Abstract 2

A self-adhesive bilayered implant mimicking the stiffness, strength, and hydration of superficial-transitional zones of articular cartilage. This design leverages triple network hydrogels with polyelectrolyte and polyampholyte networks.

## Graphical abstract

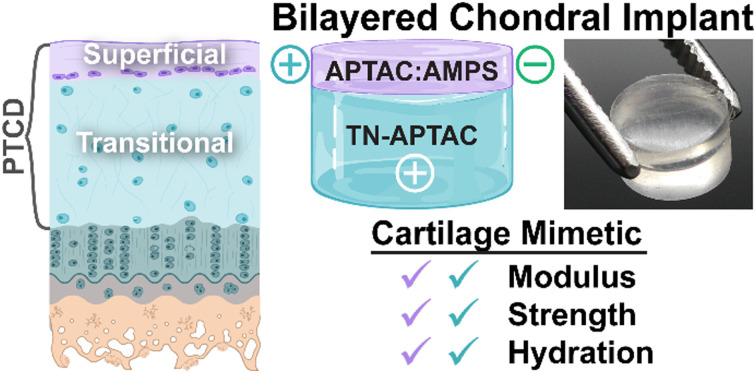

## Introduction

**Fig. 1:**
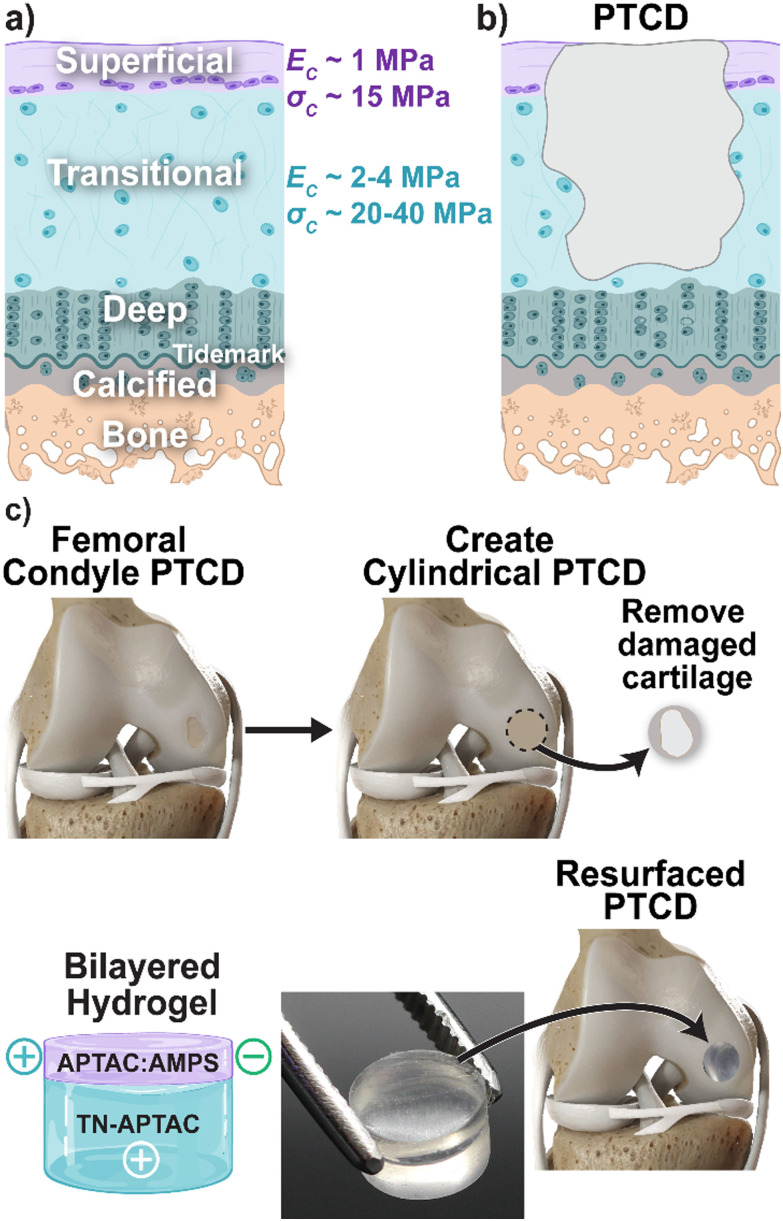
(a) Articular cartilage highlighting depth-dependent mechanical properties of the superficial and transitional deep zones, (b) depiction of partial thickness chondral defect (PTCD) with loss of superficial and transitional zones, and (c) the proposed utility of the bilayered hydrogel implant to treat a PTCD.

Articular cartilage of the knee and other joints provides low-friction motion, shock absorption, and stability.^1–3^ Its functionality stems from discrete, spatially-dependent biomechanical properties conferred by the alignment and density of collagen fibers, producing distinct compressive modulus [*E*_C_] and compressive strength [*σ*_C_] values. Three major zones comprise articular cartilage: (i) superficial [*E*_C_ ∼ 1 MPa; *σ*_C_ ∼ 15 MPa], (ii) transitional [*E*_C_ ∼ 2–4 MPa and *σ*_C_ ∼ 20–40 MPa], and (iii) deep [*E*_C_ ∼ 5 MPa and *σ*_C_ ∼ 50 MPa] zones (Fig. 1a).^1,2,4–7^ Focal chondral defects of the knee joint are extremely prevalent, with an incidence of more than 60% among patients undergoing knee arthroscopy.^8,9^ Due to the avascular nature of cartilage, these defects are unable to heal,^5,10–12^ leading to pain, loss of joint function, and eventually osteoarthritis (OA).^13–19^ Classification of these defects is based on depth: partial-thickness chondral defects (PTCDs) [reaching up to the deep layer, diameter <1.5 cm] (Fig. 1b), full thickness chondral defects (FTCDs) [reaching beyond the deep layer to calcified layer, diameter >1.5 cm], and osteochondral defects (OCDs) [reaching beyond the calcified layer to subchondral bone].^20–24^ More recently, the clinical significance of PTCDs has been noted.^20,25^ While individuals with PTCDs may be asymptomatic, knee joint pain can result and is worsened if a load-bearing region of the knee is affected. It has also been concluded that PTCDs and FTCDs of the knee contribute equally to additional cartilage damage and OA.^25,26^ Thus, PTCDs are a major contributing factor to the eventual need for a total knee replacement (TKR). Repair of PTCDs is therefore of significant consequence but necessitates the demanding recapitulation of the transitional and superficial chondral layers.

The treatment of PTCDs of the knee represents a particular challenge as, unlike FTCDs and OAs, there is a total absence of blood supply and bone marrow exudation at the defect, and surfaces contain anti-cell adhesive proteoglycans.^20,27^ The only surgical treatment specific for PTCDs is the removal of the lesions *via* chondroplasty (*i.e.*, debridement) and ablation.^23,28^ Debridement and ablation treatments have varied success rates, ranging from 30% to 70%.^23,28–32^ Numerous other surgical treatments have been broadly applied to treat the loss of articular cartilage of the knee. Cell- and tissue-based approaches such as microfracture, autologous chondrocyte implantation (ACI), and osteochondral grafting with harvested cylindrical plugs (*e.g.*, osteochondral autograft [OATS] and mosaicplasty) are commonly utilized.^33–35^ However, these are limited by a myriad of issues, including formation of fibrocartilage (mosaicplasty), cost (ACI), and donor site morbidity and delamination (OATS).^36–38^ Cartilage regeneration involving the use of a scaffold, and exogenous growth factors and/or cells has been proposed with varying success.^12,39–43^ Cartilage substitutes have emerged to provide a lubricating surface, including decellularized cartilage sheets, stem-cell loaded cellulose, and collagen meshes.^44–47^ However, the inclusion of cells and biological factors makes them expensive, reduces shelf lives, and risks an on-target response.^48–53^ Both regenerative scaffolds and biopolymer-based cartilage substitutes are also generally constrained by their inability to withstand the immediate load-bearing articulation of the knee.^10,12,38^ Thus, clinical cartilage resurfacing is performed with implants based on metal, ceramic, and hard plastic components (*e.g.*, BioPoly®, BIOLOX®, and PEEK-OPTIMA^TM^).^54–57^ The mechanical mismatch between these resurfacing devices (*e.g.*, BioPoly®: UHMWPE [*E* ∼ 200 MPa] and titanium alloy [Ti-6Al-4V] [*E* ∼ 100 GPa]; BIOLOX® ceramic [*E* ∼ 400 GPa]; and PEEK-OPTIMA^TM^ [*E* ∼ 3.5 GPa]), and the much weaker native articular cartilage (*E* ∼ 1–5 MPa) can give rise to further tissue damage.^58–62^ However, implant loosening can occur due to tissue loss stemming from ultra-high molecular weight polyethylene (UHMWPE) wear debris, as well as from stress-shielding.^33,56^ Owing to the difficulties in treating chondral defects, a total knee replacement (TKR) is often necessary.^63^

The treatment of PTCDs could be revolutionized by an off-the-shelf surgical product that could effectively recapitulate superficial and transitional zones. While conventional hydrogels may exhibit high hydration akin to native cartilage (60–90%),^64–67^ providing the potential for lubricity, they lack the requisite modulus and strength. For instance, poly(ethylene glycol) diacrylate (PEG-DA) is a broadly studied biomaterial, including cartilage regeneration,^67–69^ but lacks sufficient mechanical properties (PEG-DA 3.4 kDa; *E*_C_ ∼ 200 kPa, and *σ*_C_ ∼ 130 kPa).^70,71^ Hydrogels with substantially improved mechanical properties have emerged. J. P. Gong *et al.* reported a double network (DN) design, leveraging electrostatic repulsive charge interactions, and an asymmetric network.^72–74^ This DN was comprised of a tightly crosslinked, 1st network formed from an anionic 2-acrylamido-2-methylpropane sulfonic acid (AMPS), and a loosely crosslinked 2nd network based on neutral acrylamide (AAm). The resulting PAMPS/PAAm DN achieved ultra-high strength (*σ*_C_ ∼ 17 MPa), but a low modulus (*E*_C_ ∼ 300 kPa). In our prior work, multi-network hydrogels were formed with an anionic PAMPS 1st network, but also included hydrophobic associations as additional dynamic bonds in the 2nd network *via* the inclusion of *N*-isopropylacrylamide (NIPAAm). Means *et al.* prepared a DN wherein the loosely crosslinked 2nd network was a copolymer prepared from NIPAAm and AAm [P(NIPAAm-*co*-AAm)].^75,76^ This DN PAMPS/P(NIPAAm-*co*-AAm) produced not only ultra-high strength (*σ*_C_ ∼ 25 MPa) but a high modulus (*E*_C_ ∼ 1 MPa). Demott *et al.* produced triple networks (TN), wherein a cationic 3rd network based 3-(acrylamidopropyl)trimethyl-ammonium chloride solution (APTAC) additionally afforded intra-network electrostatic repulsive forces (within the 3rd network), and inter-network electrostatic attractive forces (between the anionic 1st network and a cationic 3rd network).^77^ A resulting TN hydrogel (TN-APTAC) exhibited both exceptional strength (*σ*_C_ ∼ 30 MPa) and modulus (*E*_C_ ∼ 3 MPa),^77^ as well as excellent lubricity.^76,78^ Additionally, these mechanically robust hydrogels were confirmed to be cytocompatible *via* lactate dehydrogenase (LDH) assays.^75,77^ While our reported DN and TN hydrogels mimicked the mechanical properties of the superficial and transitional zones of articular cartilage, respectively, their inability to be readily merged limits their potential to form a bilayered PTCD implant.

Fabrication of multilayered hydrogel constructs that mimic the regional mechanical properties of cartilage, such as the superficial and transitional zones, is a challenging task. *In situ*, multistep curing methods used to form layered hydrogels^79^ have been explored, but for a PEG system whose mechanical properties were inferior to native cartilage.^80^ Adhesive hydrogels that leverage electrostatic attractive forces may be formed from polyelectrolytes or polyampholytes. Anionic or cationic polyelectrolytes achieve adhesivity *via* electrostatic attraction to oppositely charged surfaces,^81^ while polyampholytes, having both anionic and cationic charge,^82^ can potentially adhere to either type of charged surface. The sequential curing of multi-network hydrogels leads to the charge of the final network dominating surface properties.^83–85^ Thus, adhesivity was not observed between the aforementioned TN-APTAC (cationic 3rd network) and the DN [PAMPS/P(NIPAAM-*co*-AAm)] (neutral 2nd network). However, in our subsequent studies, the TN-APTAC was adhesive to a TN hydrogel having an anionic 3rd network [PAMPS/P(NIPAAM-*co*-AAm)/PAMPS] (TN-AMPS).^86^ Yet, while the TN-AMPS exhibited a modulus (*E*_C_ ∼ 1 MPa) analogous to that of the superficial articular cartilage, it had low compressive strength (*σ*_C_ ∼ 5 MPa). Thus, to prepare a PTCD implant, a hydrogel representing the superficial zone must be fabricated to be both mechanically mimetic and adhesive to a transitional zone hydrogel (*e.g.*, TN-APTAC).

**Fig. 2:**
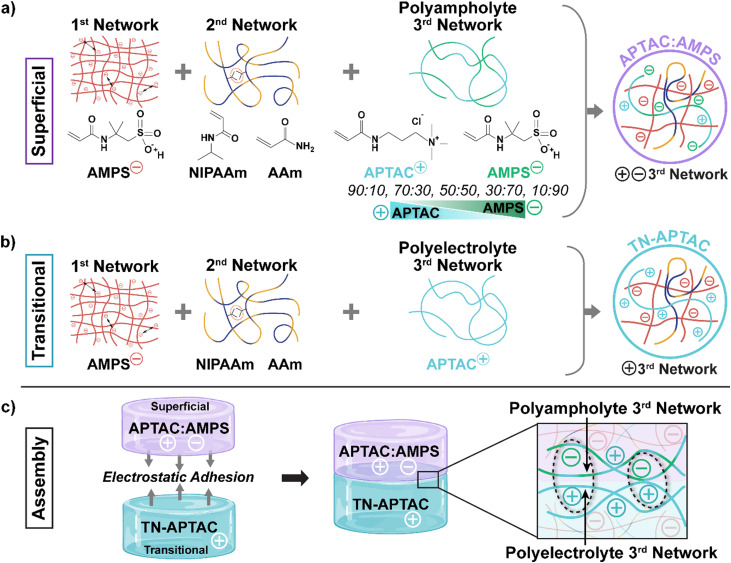
Design of a PTCD replacement: (a) superficial layer candidates: TN hydrogels formed with polyampholyte 3rd network of varying molar ratios of cationic APTAC to anionic AMPS; (b) transitional layer: TN-APTAC hydrogel with cationic 3rd network; (c) self-assembly of bilayered superficial-transitional cartilage mimetic construct *via* electrostatic attraction between charged 3rd networks.

Herein, we report a bilayered hydrogel construct that recapitulates the superficial and transitional zones of articular cartilage, and that are ‘self-assembled’ due to innate adhesivity to one another (Fig. 1c). The diameters may be readily adjusted to fit into surgically created, drilled defects within lesions, as with autografting. To do so, a hydrogel was developed to serve as the superficial layer, with mimetic mechanical properties as well as adhesivity to the transitional layer hydrogel (TN-APTAC). Superficial layer candidates were formed as TN hydrogels with a polyampholyte 3rd network (Fig. 2). The molar ratio of cationic APTAC to anionic AMPS in the 3rd network was systematically tuned (*i.e.*, 90 : 10, 70 : 30, 50 : 50, 30 : 70, and 10 : 90 molar ratio). The charge character of the polyampholyte 3rd network was anticipated to have competing effects. A more anionic 3rd network was expected to produce TN hydrogels with greater adhesivity, *via* electrostatic attraction, to the cationic surface of TN-APTAC. Yet, an increasingly anionic 3rd network would diminish rigidity and strength due to greater inter-network repulsion with the anionic 1st network, leading to greater swelling. The effect of crosslink density of the polyampholyte 3rd network was also explored by using two different crosslinking levels by varying the crosslinker content. Compared to the highly crosslinked 1st network, both 3rd network crosslink densities were relatively loosely crosslinked as this was expected to avoid brittleness. However, varying the 3rd network crosslink density was hypothesized to potentially impact charge mobility, and hence mechanical properties and adhesivity.

## Experimental

### Materials

Acrylamide (AAm, >99%), 2-acrylamido-2-methylpropane sulfonic acid (AMPS, 97%), (3-acrylamidopropyl)trimethyl-ammonium chloride solution (APTAC; 75 wt% in H_2_O), *N*,*N*′-methylene-bis-acrylamide crosslinker (BIS, 99%), *N*-isopropyl-acrylamide (NIPAAm; 97%), 2-oxoglutaric acid photoinitiator (2-oxo, 99.0–101.0%), were obtained from Millipore-Sigma. Deionized (DI) water (18.2 MΩ cm, Thermo Scientific^TM^ Barnstead^TM^ E-Pure^TM^ Ultrapure Water Purification System) was used for hydrogel fabrication. Water-resistant 60 grit sandpaper and ⅛” × ½” (thickness × width) multipurpose 6061 aluminum bars were purchased from McMaster Carr.

### Triple network (TN) hydrogel fabrication

TN hydrogels were fabricated through a multi-step process in which each network was subsequently UV cured. The hydrogels were cured beginning with a single network (SN) precursor solution formulated from AMPS (1.5 M), BIS crosslinker (4 mol% w.r.t. AMPS), and 2-oxo photoinitiator (0.1 mol% w.r.t. AMPS) in DI water. A custom mold was created consisting of glass slides separated by silicone spacers (∼1 mm thickness), the solution was cured in the molds on a UV plate (UV-transilluminator, 6 mW cm^−2^, 365 nm) for 5 hour, flipping the mold every 15 min for the first hour and on the hour for 4 subsequent hours to promote uniformity. The SN cured hydrogels’ edges were trimmed then submerged in the double network (DN) precursor solution. When initially submerging the SN hydrogels in the precursor solution, the surface was irrigated with the DN solution to avoid curling and fracture of the SN hydrogels. DN solution was formulated from NIPAAm (2.0 M), AAm (10 wt% w.r.t. NIPAAm), BIS (0.10 mol% w.r.t. NIPAAm), and 2-oxo (0.10 mol% w.r.t. NIPAAm) in DI water. Following submersion for 48 hour, the hydrogels were UV-cured in glass molds separated by polycarbonate spacers (∼1 mm thickness) for 5 hour in an ice bath (∼7 °C), following the SN hydrogel ‘flipping pattern’. Cured DN hydrogels were soaked in triple network (TN) precursor solutions composed of a total monomer concentration of 2.0 M. The TN polyampholyte (PA) 3rd network precursor solution was fabricated with varied molar ratios of cationic APTAC to anionic AMPS denoted *X* : *Y*, (*e.g.*, 90 : 10, 1.8 M APTAC to 0.2 M AMPS). In addition, TN hydrogels were likewise formed with either a cationic-only (TN-APTAC) or anionic-only (TN-AMPS) 3rd network with a monomer concentration of 2.0 M.^86^ In addition to the monomers, BIS crosslinker (0.10 mol% or 0.05 mol% w.r.t. total monomer concentration in the designated network) and 2-oxo photoinitiator (0.10 mol% w.r.t. total monomer concentration in the designated network) were added to the 3rd network precursor solution. Following 48 hour soaking hydrogels were UV-cured as above. The resulting TN hydrogels were removed from the mold and placed in DI water for at least 7 days prior to testing described below.

### Methods

#### Equilibrium water content (EWC)

EWC of the TN hydrogels was determined from the swollen and dry masses of disc specimens. Hydrogel discs (*N* ≥ 5) were punched with a 6 mm biopsy punch, and surface water was removed by blotting the surface with a Kim Wipe. Discs were placed in an oven at 60 °C and dried for 12 hour under vacuum (30 in. Hg). Water content was calculated by taking the swollen mass (*M*_w_) and dry mass (*M*_d_), then calculating water content as 
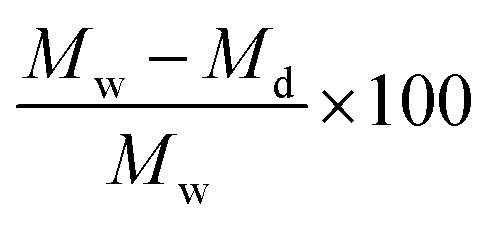
.

#### ‘Post-cure’ mass swelling and diameter increase

Immediately following TN hydrogel curing, samples (*N* ≥ 3) were punched from sheets using a 6 mm biopsy punch, and the initial diameters (*D*_c_) and masses (*M*_c_) were immediately recorded. Hydrogel discs were then immersed in DI water for 7 days. Swollen hydrogel discs were then blotted with a Kim Wipe to remove surface water, and swollen masses (*M*_s_) and diameters (*D*_s_) were recorded. Hydrogel post-cure mass swelling was calculated by 
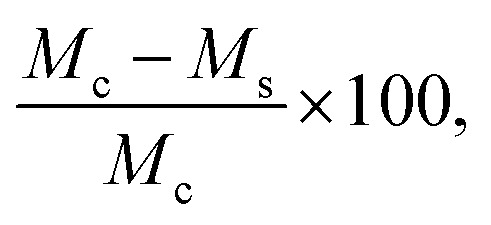
 and post-cure diameter increase was calculated by 
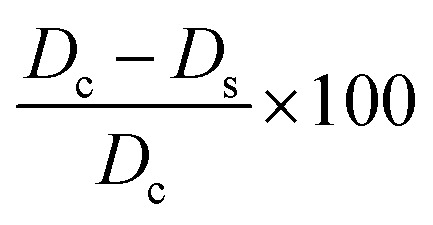
.

#### Compression testing

Unconfined compressive mechanical properties of the TN hydrogels were evaluated using an Instron 5944 in ambient conditions. TN hydrogel samples (*N* ≥ 5) were punched using a 6 mm biopsy punch, and surface water was blotted off with a Kim Wipe off just prior to testing. Samples were preloaded with a 0.2 N force and strain was zeroed. Samples were compressed at a rate of 1 mm min^−1^ until fracture. Compressive modulus values were calculated from designated slopes of linear regions (0–10% strain, 40–50%, and 70–80%). Compressive strength was designated as the stress at the point of fracture, and ultimate compressive strain corresponded to the strain at fracture. Compressive toughness was calculated from the area under the stress–strain curve prior to fracture.

#### Dynamic mechanical analysis (DMA)

Viscoelastic mechanical properties (*e.g.*, loss modulus, storage modulus, tan *δ*) of the TN hydrogels were evaluated using a TA Instruments DMA Q800. Specimens (*N* ≥ 5) were prepared following parallel protocol to the unconfined compression specimens. Testing was conducted under a multi-frequency strain mode from 1–30 Hz at an amplitude of 10 μm with a preload of 0.1 N. Values reported from 10 Hz, in which tan *δ* was calculated with the loss (*G*′) and storage modulus (*G*′′), 
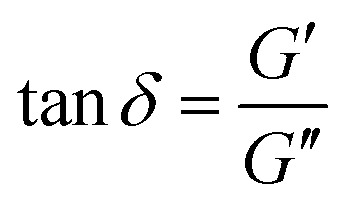
.

#### Tensile testing

Tensile mechanical properties of the TN hydrogels were evaluated using an Instron 5944 in ambient conditions. TN hydrogel bone specimens (*N* ≥ 3) were punched using a certified dog bone punch (ASTM D1708-18).^87^ Surface water was blotted with a Kim Wipe just prior to testing. Sandpaper was attached to tension clamps to prevent specimen slippage during testing. Samples were preloaded with a 0.2 N force, and strain was zeroed. Samples were elongated at a rate of 10 mm min^−1^ until fracture. The tensile modulus was defined as the slope of the linear region (0–10% strain). Tensile strength and ultimate tensile strain were defined as the stress and strain at the point of fracture, respectively. Tensile toughness was determined by area under the stress–strain curve prior to fracture.

#### Qualitative adhesion testing

Hydrogel specimens [5 mm × 10 mm (width × length)] were blotted with a Kim Wipe to remove surface water and aligned to have a 5 mm × 5 mm (width × length) contact area (*i.e.*, connection). Pressure was applied by hand to the connection for 60 s. Qualitative adhesion was immediately evaluated in terms of the response of the connection when the construct was orientated by hand vertically (*i.e.*, along a tensile axis): (i) no adhesion [*N*] (*i.e.*, no adherence by the connection), (ii) slight adhesion [*G*] (*i.e.*, the connection could only withstand gravity), (iii) adhesive failure [*A*] (*i.e.*, when tension applied by hand, the connection fails), and (iv) cohesive failure [*C*] (*i.e.*, when tension applied by hand, weaker hydrogel fails prior connection). In this way, adhesivity of the connection increased as follows: *N* < *G* < *A* < *C*.

#### Lap shear testing

The adhesivity of TN hydrogels was quantitatively measured *via* lap shear testing. A custom mold was employed to ensure constistent alignment and contract area among adhered hydrogel specimens per a previous report.^86^ Rectangular specimens [1 cm × 4 cm (width × length)] were first blotted with a Kim Wipe to remove surface water, and then placed in the custom mold to ensure a 1 cm^2^ contact area (*i.e.*, connection). Pressure was subsequently applied by hand to the connection for 1 min. The resulting constructs were removed from the mold, and soaked in DI water for 48 hour. Constructs (*N* ≥ 3) were subjected to lab shear testing under ambient conditions. A modified lap shear set-up was employed,^86^ wherein aluminum supports were added to the upper and lower clamps to maintain alignment hydrogel specimens. These supports were fabricated from ⅛ inch thick aluminum bars (McMaster Carr) cut to a length of 2.75 inches. Each support and tension clamp had sandpaper attached to prevent hydrogel slippage during testing. A length of 1 cm of the rectangular hydrogel construct was secured into the clamp and aligned by the support. Next, a preload of 0.2 N was applied and strain was zeroed. Specimens were elongated at a rate of 10 mm min^−1^ until failure. Shear strength was defined as the stress at the point of failure of the interface or fracture of hydrogel. Shear strength was calculated based on the area of the connection (1 cm^2^).

### Statistical analysis

One-way and two-way ANOVA with Tukey's multiple comparisons tests were used to statistically analyze all data sets. Analyses were conducted with GraphPad Prism (Version 9.4.0) using a standard α level of 0.05. All comparisons with a *p* < 0.05 was statistically significant.

## Results and discussion

### Hydrogel fabrication

A series of TN hydrogels with a polyampholyte 3rd network were fabricated as potential superficial layer candidates with mimetic mechanical properties and adhesivity to the transitional layer (TN-APTAC) (Fig. 2 and Table S1, ESI†). The 3rd network charge composition was tuned based on the molar ratio of cationic APTAC to anionic AMPS (90 : 10, 70 : 30, 50 : 50, 30 : 70, and 10 : 90). Given the dominance of the final network charge of multi-network hydrogels,^83–85^ 90 : 10 and 70 : 30 would possess a cationic-dominant surface, 30 : 70 and 10 : 90 an anionic-dominant surface, and 50 : 50 a ‘charge-balanced’ surface. In addition to the TN-APTAC having a cationic-only 3rd network, TN-AMPS was formed with an anionic-only 3rd network as a control. TN hydrogels with a more anionic polyampholyte 3rd network (*i.e.*, increased AMPS) would exhibit increased inter-network electrostatic repulsion with the anionic 1st network while those with more cationic 3rd networks (*i.e.*, increased APTAC) would exhibit increased inter-network attraction. All TN hydrogels were fabricated through a multi-step, sequential curing process in which the 1st network was composed of tightly crosslinked anionic PAMPS (4 mol% BIS), and the 2nd network was loosely crosslinked and neutral P(NIPAAm-*co*-AAm) (0.1 mol% BIS). Thus, the 1st network exhibited intra-network repulsive forces, and the 2nd network exhibited intra-network hydrophobic associations. These aforementioned interactions effectively served as dynamic crosslinks to impart robust mechanical properties. The 3rd network total monomer concentration (2.0 M) was maintained across all TN hydrogel compositions, and was selected to be used based on TN-APTAC as it was previously noted to produce a plateau in compressive modulus (*E*_C_ ∼ 3 MPa) and strength (*σ*_C_ ∼32 MPa).^77^ The 3rd network crosslink density was varied by employing the crosslinker (BIS) at two levels (0.10 or 0.05 mol% BIS), but both were relatively loosely crosslinked *versus* the 1st network. The higher crosslinker level (*i.e.*, 0.10 mol% BIS) represents that used to prepare the 3rd network of the TN-APTAC transitional layer.^77^ A reduction in crosslinking (*i.e.*, 0.05 mol% BIS) was considered to potentially increase chain and charge mobility of the 3rd network.

### Hydrogel water content and swelling

**Fig. 3:**
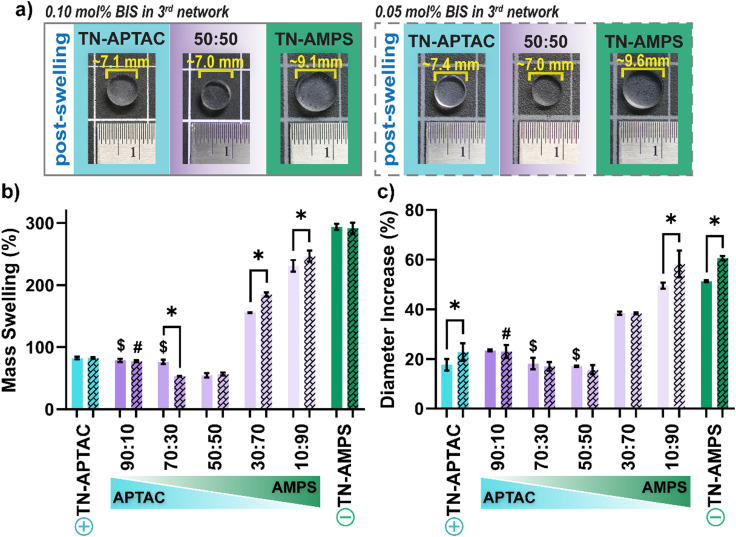
Post-swelling behavior TN hydrogels: (a) swelling photo series of select compositions, (b) mass swelling (%), and (c) increase in diameter (%). Solid bars represent TNs with a 3rd network prepared with 0.10 mol% BIS, and dashed bars represent TNs with a 3rd network prepared with 0.05 mol% BIS. * *p* < 0.05 for TN hydrogels (0.10 mol% BIS) *vs.* TN hydrogels (0.05 mol% BIS); $ *p* > 0.05 for TN-APTAC (0.10 mol% BIS) *vs.* TN hydrogels (0.10 mol% BIS); and # *p* > 0.05 for TN-APTAC (0.05 mol% BIS) *vs.* TN hydrogels (0.05 mol% BIS). (Results for all TN hydrogels shown in Fig. S2 and Table S3, ESI†).

Hydrogel hydration is an essential property in terms of both cartilage mimicry and impact on mechanical properties.^88^ The effect of 3rd network charge composition on TN hydrogel properties was thoroughly assessed. In terms of gravimetric water content, both the TN-APTAC and the TN-AMPS exhibited values between 74 and 90 wt%, within the range of cartilage tissue (60–90 wt%) (Fig. S1 and Table S2, ESI†). TNs with a polyampholyte 3rd network likewise produced water content values in this range, irrespective of charge composition or crosslink density (*i.e.*, 0.10 or 0.05 mol% BIS) of the 3rd network. Hydration was further assessed in terms of swelling behaviour (*via* mass swelling and diameter increase) following equilibration of freshly cured specimens (initial diameter ∼6 mm) in DI water (Fig. 3, Fig. S2 and Table S3, ESI†). Likely due to electrostatic repulsion with the anionic 1st network,^72,77,86^ as the 3rd network became more anionic (*i.e.*, increased AMPS), swelling increased. This led to notable increases in post-swelling mass and diameter. For instance, for TN hydrogels prepared with a relatively higher crosslink density 3rd network (*i.e.*, 0.10 mol% BIS), swollen mass increased as follows: ∼55% (50 : 50) < ∼155% (30 : 70), < ∼230% (10 : 90) < ∼300% (TN-AMPS). Overall, swelling was not substantially changed for analogous TN hydrogels prepared with a reduced crosslink density of the 3rd network (*i.e.*, 0.05 mol% BIS) (Fig. 3, Fig. S2 and Table S3, ESI†). This result is attributed to both 0.10 and 0.05 mol% BIS producing rather loosely crosslinked 3rd networks, particularly as compared to the tightly crosslinked 1st network that was prepared with 4.0 mol% BIS.

### Hydrogel mechanical properties

**Fig. 4:**
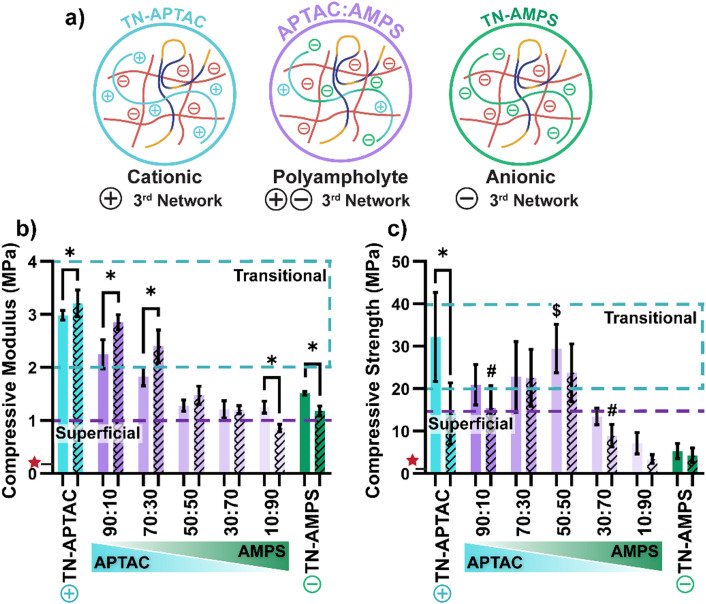
Compressive mechanical properties of TN hydrogels: (a) representative schematics for TN hydrogel compositions, (b) compressive modulus, and (c) compressive strength. Solid bars represent TNs with a 3rd network prepared with 0.10 mol% BIS, and dashed bars represent TNs with a 3rd network prepared with 0.05 mol% BIS. Blue dashed region highlights native-like values of transitional cartilage, and purple dashed line highlights native-like values of superficial cartilage.^1,4–6^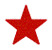 Denotes PEG-DA (3.4 kDa, 10 wt%) mechanical properties.^70^ * *p* < 0.05 for TN hydrogels (0.10 mol% BIS) *vs.* TN hydrogels (0.05 mol% BIS); $ *p* > 0.05 for TN-APTAC (0.10 mol% BIS) *vs.* TN hydrogels (0.10 mol% BIS); and # *p* > 0.05 for TN-APTAC (0.10 mol% BIS) *vs.* TN hydrogels (0.05 mol% BIS).^77,86^ (Compressive mechanical properties also shown in Fig. S3, Fig. S4 and Table S4, ESI†).

To serve as a cartilage substitute, a hydrogel must mimic the mechanical properties of the native superficial layer, including stiffness and strength. Towards identifying a TN hydrogel having a polyampholyte 3rd network that can serve as the superficial layer, unconfined compression tests were utilized to assess the effect of 3rd network charge composition as well as crosslink density (Fig. 4, Fig. S3, Fig. S4 and Table S4, ESI†). PEG-DA (3.4 kDa) hydrogels have been extensively studied as a biomaterial for a variety of applications including cartilage; however, its limited mechanical properties (*e.g.*, *E*_C_ ∼200 kPa and *σ*_C_ ∼130 kPa) make it not suitable for immediate load-bearing.^70,71^ For all TN hydrogels, the compressive modulus and strength values far exceed that of PEG-DA (note: PEG-DA indicated with a red star [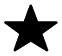] in Fig. 4). Overall, compressive modulus (*E*_C_) values of TN hydrogels decreased when the 3rd network became more anionic (*i.e.*, increased AMPS). This was largely attributed to increased swelling and hydration, stemming from electrostatic repulsion between the anionic 1st and 3rd networks. Among TN hydrogels formed with a higher crosslinked density 3rd network (*i.e.*, 0.10 mol% BIS), *E*_C_ values decreased from TN-APTAC (*E*_C_ ∼ 3.0 MPa) to TN-AMPS (*E*_C_ ∼ 1.5 MPa). A reduction of crosslink density of the 3rd network (*i.e.*, 0.05 mol% BIS), generally led to somewhat of an increase in *E*_C_ for TN hydrogels, particularly when the 3rd network was more cationic. A transitional layer-like *E*_C_ was achieved by not only TN-APTAC [0.10 mol% BIS], but also by TN-APTAC [0.05 mol% BIS] (*E*_C_ ∼ 3.2 MPa), 90 : 10 [0.10 mol% BIS] (*E*_C_ ∼2.3 MPa), 90 : 10 [0.05 mol% BIS] (*E*_C_ ∼ 2.9 MPa), and 70 : 30 [0.05 mol% BIS] (*E*_C_ ∼ 2.4 MPa). In terms of a superficial layer-like *E*_C_, several TN hydrogels with polyampholyte 3rd networks were mimetic: 50 : 50, 30 : 70, and 10 : 90 (each at 0.10 mol% and 0.05 mol% BIS). Their similarity in *E*_C_ (∼1.3 MPa) is despite a notably increased swelling as the 3rd network became more anionic, suggesting that swelling-induced strain stiffening may have contributed. While strains >10% exceed the physiological range of normal or impact loading, modulus of TN hydrogels were also evaluated at higher strains as typical for robust hydrogels (Table S4, ESI†).^89,90^ An increase in the slopes of stress *versus* strain curves at higher strains were notably apparent (Fig. S4, ESI†). These were associated with marked increases in stiffness due to strain hardening effects, with modulus values increasing to ∼5–11 MPa (40–50% strain) and ∼26–73 MPa (70–80% strain). The moduli at 70–80% strain of certain TNs hydrogels with highly anionic 3rd networks [TN-AMPS (0.10 mol% and 0.05 mol% BIS) and 10 : 90 (0.10 mol% BIS)] could not be determined as they fractured just prior to ∼70% strain.

In terms of compressive strength (*σ*_C_), TN-APTAC [0.10 mol% BIS] displayed an impressive value (*σ*_C_ ∼ 32 MPa), within the range of transitional cartilage. In contrast, the strength of TN-AMPS [0.10 mol% BIS] was notably decreased (*σ*_C_ ∼ 5 MPa) to well below that of superficial cartilage. For TN hydrogels with a polyampholyte 3rd network (0.10 mol% BIS), *σ*_C_ generally decreased with greater anionic charge of the 3rd network to a minimum of *σ*_C_ ∼7 MPa (10 : 90). As 3rd network crosslink density was decreased (0.05 mol% BIS), there was a decrease in *σ*_C_ that can also be related to an increased swelling. This reduction was dramatic for TN-APTAC [0.05 mol% BIS] whose *σ*_C_ was reduced to ∼14 MPa, below that of transitional cartilage. A transitional layer-like *σ*_C_ was achieved by not only TN-APTAC [0.10 mol% BIS], but also 90 : 10 [0.10 mol% BIS] (*σ*_C_ ∼21 MPa), 90 : 10 [0.05 mol% BIS] (*σ*_C_ ∼15 MPa), 70 : 30 [0.10 mol% BIS] (*σ*_C_ ∼23 MPa), 70 : 30 [0.05 mol% BIS] (*σ*_C_ ∼23 MPa), 50 : 50 [0.10 mol% BIS] (*σ*_C_ ∼30 MPa), and 50 : 50 [0.05 mol% BIS] (*σ*_C_ ∼24 MPa). In contrast, only 30 : 70 (0.10 mol% BIS) achieved superficial layer-like *σ*_C_ (∼13 MPa).

All TNs were notably non-brittle, as marked by ultimate strain at fracture values ranging from ∼70 to ∼90%, and toughness values that ranged from ∼1.0 to ∼4.5 MJ m^−3^ (Table S4, ESI†). In terms of viscoelasticity, native articular cartilage exhibits an elastic response to cyclical loading.^91^ Likewise, when evaluated *via* compressive dynamic mechanical testing, all TNs demonstrated a dominant elastic response (tan *δ* < 1) (Fig. S5 and Table S5, ESI†). While articular cartilage experiences compressive loading, TN hydrogel modulus and strength was also assessed under tension, and similar trends were observed (Fig. S6, Fig. S7 and Table S6, ESI†). When the 3rd network was prepared with the higher crosslink density (0.10 mol% BIS), the tensile strength (*σ*_T_) increased from ∼1.1 MPa (TN-APTAC) to ∼1.4 MPa (90 : 10, 70 : 30, and 50 : 50), then decreased to ∼0.8 MPa (30 : 70 and 10 : 90), and further down to ∼0.6 MPa (TN-AMPS). The tensile properties were not substantially altered when the crosslink density of the 3rd network was reduced (0.05 mol% BIS).

Overall, achievement of both mimetic *E*_C_ (strain ≤10%) and *σ*_C_ was achieved by specific TN hydrogels. For the transitional layer, this was limited to TN-APTAC [0.10 mol% BIS] as well as 90 : 10 [0.10 mol% BIS] and 70 : 30 [0.05 mol% BIS], while for the superficial layer, this was limited to 30 : 70 [0.10 mol% BIS]. Mechanical mimicry to the surrounding tissue is critical to healing and to the host-tissue response, as mismatch results in unequal strain responses to applied loads that can cause further tissue deterioration.^92–94^

### Adhesivity

To create a self-assembled, PTCD bilayered implant, a superficial layer hydrogel must robustly adhere to the transitional layer hydrogel. Initially, to provide broad insight to TN hydrogel adhesivity, all compositions were screened *via* qualitative tests. Rectangular specimens (5 mm × 10 mm) were blotted of surface water, contact made, and pressure applied by hand in the overlapping contact area (5 mm × 5 mm) for 60 seconds. Upon immediately orientating vertically (*i.e.*, along a tensile axis), the observed results were assigned to one of four categories: (i) no adhesion (*i.e.*, no adherence by the connection), (ii) slight adhesion (*i.e.*, the connection could only withstand gravity), (iii) adhesive failure (*i.e.*, when tension applied by hand, the connection fails), and (iv) cohesive failure (*i.e.*, when tension applied by hand, the weaker hydrogel fails prior connection) (Table S7, ESI†). The 50 : 50 [0.10 mol% and 0.05 mol% BIS] was notably neither adhesive to the cationic TN-APTAC nor the anionic TN-AMPS. This indicates that the charge balance within the 3rd network of 50 : 50 limited electrostatic attraction to the contacting surface. The desired cohesive failure was notably observed between TN hydrogels that leveraged electrostatic attractive forces between oppositely charged surfaces. This occurred for TN hydrogel pairs wherein one had an anionic-only 3rd network (*i.e.*, TN-AMPS) or an anionic-dominant polyampholyte 3rd network (*i.e.*, 70 : 30 and 90 : 10), and the other had a cationic-only 3rd network (*i.e.*, TN-APTAC) or a cationic-dominant polyampholyte 3rd network (*i.e.*, 70 : 30 and 90 : 10). This demonstrated efficacy of a polyampholyte 3rd network to establish cohesive adhesion, despite a lack of a singular charge type, is notable.

**Fig. 5:**
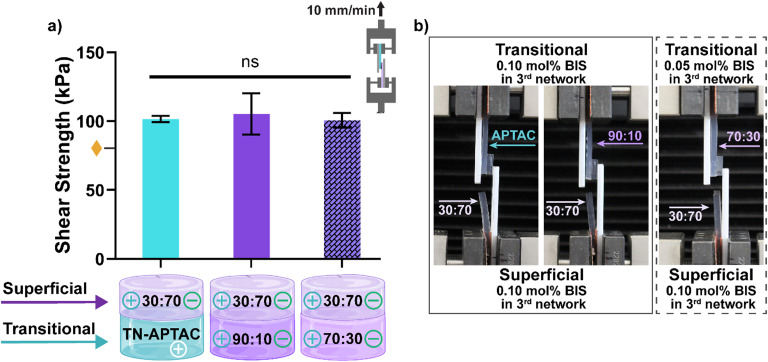
Lap shear testing of TN hydrogels representing the superficial and transitional layers: (a) shear strength at fracture: representative image of cohesive fracture of (b) 30 : 70 (0.10 mol% BIS) paired with either TN-APTAC (0.10 mol% BIS), 90 : 10 (0.10 mol% BIS), or 70 : 30 (0.05 mol% BIS). Solid bars represent (0.10 mol% BIS) in transitional layers and dashed bar represents (0.05 mol% BIS) in transitional layer. 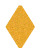 denotes lap shear strength between TN-APTAC and TN-AMPS.^86^ ns *p* > 0.05 *vs.* all compositions. (Results shown in Table S8, ESI†).

Based on compressive mechanical properties, potential transitional layers included TN-APTAC [0.10 mol% BIS], 90 : 10 [0.10 mol% BIS], and 70 : 30 [0.05 mol% BIS], while the potential superficial layer was limited to 30 : 70 [0.10 mol% BIS]. These three pairs, representative of potential PTCD bilayered constructs, demonstrated cohesive failure in the qualitative assessment. Thus, they were subsequently subjected to lap shear tests to quantify adhesion. For lap shear testing, constructs were formed by connecting the transitional layer candidates each to the superficial layer. Planar hydrogel strips (10 mm × 40 mm) which were blotted, pairs placed in contact with a 1 cm^2^ overlap, and the construct subjected to tensile strain (Fig. S8, ESI†). Lap shear strength was calculated at the point of fracture (Fig. 5 and Table S8, ESI†). Cohesive failure was likewise observed during this test, with failure occurring within the 30 : 70 [0.10 mol% BIS] owing to its relative lower tensile strength (*σ*_T_ ∼ 0.85 MPa) *versus* the adhesivity of the connection. All pairs exhibited robust lap shear strengths of ∼100 kPa. These values are even higher that the lap shear strength (∼80 kPa) of a TN-APTAC and TN-AMPS construct.^86^ However, this may be attributed to the greater tensile strength of the 30 : 70 [0.10 mol% BIS] *versus* TN-AMPS (*σ*_T_ ∼0.6 MPa) that allows the former to withstand greater force during the lap shear test. Overall, the 30 : 70 [0.10 mol% BIS] superficial layer was able to form robust adhesion to all three potential transitional layers (TN-APTAC [0.10 mol% BIS], 90 : 10 [0.10 mol% BIS], and 70 : 30 [0.05 mol% BIS]).

## Conclusions

Towards treating PTCDs, a desirable approach is a bilayered hydrogel implant that recapitulates the regional mechanical properties of the superficial and transitional chondral layers and can be readily assembled. This work demonstrates an approach to achieve this by leveraging TN hydrogels prepared with a polyampholyte 3rd network. Inter- and intra-network electrostatic as well as hydrophobic associations were leveraged as dynamic bonds to impart mechanical robustness and surface adhesivity. All TN hydrogels were formed with an anionic, highly crosslinked 1st network [“PAMPS”], and a neutral, loosely crosslinked 2nd network crosslinked 2nd network [P(NIPAAm-*co*-AAm)]. Thus, the 1st and 2nd networks produce intra-network electrostatic repulsion and hydrophobic associations, respectively. TN hydrogels with a polyampholyte 3rd network were formed with 5 charge compositions by varying the molar ratio of cationic [APTAC] to anionic [AMPS] monomer: 90 : 10, 70 : 30, 50 : 50, 30 : 70, and 10 : 90 molar ratio. TNs with an exclusively cationic [TN-APTAC] and anionic [TN-AMPS] 3rd networks were also formed as controls. Additionally, for each composition, the 3rd network was formed with 0.10 mol% or 0.05 mol% BIS to slightly tune crosslink density of the loosely crosslink 3rd network. Thus, the 3rd network introduces intra-network electrostatic interactions as well as inter-network electrostatic interactions with the anionic 1st network. All TN hydrogels exhibited cartilage-like hydration (>74%). As the 3rd network became more anionic (*i.e.*, increased AMPS), TN hydrogels exhibited greater swelling marked by a larger specimen diameter increase. TN hydrogels with mimetic compressive modulus and strength were identified. For the transitional layer, this was achieved by TN-APTAC [0.10 mol% BIS] as well as 90 : 10 [0.10 mol% BIS] and 70 : 30 [0.05 mol% BIS], whereas for the superficial layer, this was limited to 30 : 70 [0.10 mol% BIS]. The capacity of the superficial layer and transitional layer candidates to be joined was evaluated. When connected *via* simple pressing together by hand, these TN hydrogel pairs exhibited robust adhesion in both qualitative adhesion tests and in lab shear tests. Specifically, lap shear strengths of ∼100 kPa were demonstrated by all three constructs, with failure occurring away from the connection (*i.e.*, cohesive failure rather than adhesive failure). This result is attributed to the dominance of the 3rd network charge composition on surface properties. It demonstrates that the surface charge does not need to be exclusively cationic and anionic to give rise to adhesivity, but may also be achieved by polyampholyte 3rd network having a ‘cationic-dominant’ and ‘anionic-dominant’ charge composition. Overall, this approach represents robust strategy to constructing PTCD implants with mimetic regional mechanical properties of the superficial and transitional layers.

## Author contributions

The manuscript was written through contributions of all authors. All authors have given approval to the final version of the manuscript.

## Data availability

Raw data is available *via* the Texas Data Repository (TDR).

## Conflicts of interest

There are no conflicts to declare.

## Supplementary Material

TB-013-D5TB00050E-s001

## References

[cit1] Sophia Fox A. J., Bedi A., Rodeo S. A. (2009). Sports Health.

[cit2] Chen S. S., Falcovitz Y. H., Schneiderman R., Maroudas A., Sah R. (2001). Osteoarthr. Cartil..

[cit3] Bhosale A. M., Richardson J. B. (2008). Br. Med. Bull..

[cit4] Petitjean N., Canadas P., Royer P., Noël D., Le Floc'h S. (2022). J. Biomed. Mater. Res., Part A.

[cit5] Camarero-Espinosa S., Rothen-Rutishauser B., Foster E. J., Weder C. (2016). Biomater. Sci..

[cit6] Sadeghi H., Espino D. M., Shepherd D. E. T. (2017). BMC Musculoskeletal Disord..

[cit7] Wilson W., Huyghe J., Van Donkelaar C. (2007). Biomech. Model. Mechanobiol..

[cit8] Houck D. A., Kraeutler M. J., Belk J. W., Frank R. M., McCarty E. C., Bravman J. T. (2018). Orthop. J. Sports Med..

[cit9] Widuchowski W., Widuchowski J., Trzaska T. (2007). Knee.

[cit10] Redman S., Oldfield S., Archer C. (2005). Eur. Cells Mater..

[cit11] Verwoerd-Verhoef H. L., ten Koppel P. G. J., van Osch G. J. V. M., Meeuwis C. A., Verwoerd C. D. A. (1998). Int. J. Pediatr. Otorhinolaryngol..

[cit12] Huey D. J., Hu J. C., Athanasiou K. A. (2012). Science.

[cit13] Khan I. M., Gilbert S. J., Singhrao S. K., Duance V. C., Archer C. W. (2008). Eur. Cells Mater.

[cit14] Barrie H. (1987). J. Bone Jt. Surg., Br. Vol..

[cit15] Gelber A. C., Hochberg M. C., Mead L. A., Wang N. Y., Wigley F. M., Klag M. J. (2000). Ann. Intern. Med..

[cit16] Felson D. T., Zhang Y. (1998). Arthritis Rheum..

[cit17] Guettler J. H., Demetropoulos C. K., Yang K. H., Jurist K. A. (2004). Am. J. Sports Med..

[cit18] Caldwell K. L., Wang J. (2015). Osteoarthr. Cartil..

[cit19] Vannini F., Spalding T., Andriolo L., Berruto M., Denti M., Espregueira-Mendes J., Menetrey J., Peretti G. M., Seil R., Filardo G. (2016). Knee Surg. Sports Traumatol. Arthrosc..

[cit20] Sun D., Liu X., Xu L., Meng Y., Kang H., Li Z. (2022). Int. J. Nanomed..

[cit21] Men Y. T., Jiang Y. L., Chen L., Zhang C. Q., Ye J. D. (2017). Mater. Sci. Eng., C.

[cit22] Sellards R. A., Nho S. J., Cole B. J. (2002). Curr. Opin. Rheumatol..

[cit23] Makhni E. C., Stone A. V., Ukwuani G. C., Zuke W., Garabekyan T., Mei-Dan O., Nho S. J. (2017). Clin. Sports Med..

[cit24] Husen M., Custers R. J. H., Hevesi M., Krych A. J., Saris D. B. F. (2022). J. Cartil. Jt. Preserv.

[cit25] Guermazi A., Hayashi D., Roemer F. W., Niu J., Quinn E. K., Crema M. D., Nevitt M. C., Torner J., Lewis C. E., Felson D. T. (2017). Arthritis Rheumatol..

[cit26] Ono Y., Akagi R., Mikami Y., Shinohara M., Hosokawa H., Horii M., Watanabe S., Ogawa Y., Sadamasu A., Kimura S., Yamaguchi S., Ohtori S., Sasho T. (2021). Cartilage.

[cit27] Lewandowska K., Choi H. U., Rosenberg L. C., Zardi L., Culp L. A. (1987). J. Cell Biol..

[cit28] Lin C., Deng Z., Xiong J., Lu W., Chen K., Zheng Y., Zhu W. (2021). Front. Bioeng. Biotechnol..

[cit29] Abram S. G. F., Palmer A. J. R., Judge A., Beard D. J., Price A. J. (2020). BMJ Open.

[cit30] Fu F. H., Zurakowski D., Browne J. E., Mandelbaum B., Erggelet C., Moseley J. B., Anderson A. F., Micheli L. J. (2005). Am. J. Sports Med..

[cit31] Grieshober J. A., Stanton M., Gambardella R. (2016). Sports Med. Arthrosc. Rev..

[cit32] Krych A. J., Saris D. B. F., Stuart M. J., Hacken B. (2020). J. Am. Acad. Orthop. Surg..

[cit33] Farr J., Cole B., Dhawan A., Kercher J., Sherman S. (2011). Clin. Orthop. Relat. Res..

[cit34] Case J. M., Scopp J. M. (2016). Sports Med. Arthrosc. Rev..

[cit35] Goldring S. R., Goldring M. B. (2016). Nat. Rev. Rheumatol..

[cit36] Cong B., Sun T., Zhao Y., Chen M. (2023). Ther. Clin. Risk Manage..

[cit37] Marlovits S., Zeller P., Singer P., Resinger C., Vécsei V. (2006). Eur. J. Radiol..

[cit38] Medvedeva E. V., Grebenik E. A., Gornostaeva S. N., Telpuhov V. I., Lychagin A. V., Timashev P. S., Chagin A. S. (2018). Int. J. Mol. Sci..

[cit39] Ge Z., Li C., Heng B. C., Cao G., Yang Z. (2012). J. Biomed. Mater. Res., Part A.

[cit40] Jelodari S., Ebrahimi Sadrabadi A., Zarei F., Jahangir S., Azami M., Sheykhhasan M., Hosseini S. (2022). BioMed. Res. Int..

[cit41] Cao Z., Dou C., Dong S. (2014). J. Nanomater..

[cit42] Shah S. S., Mithoefer K. (2020). Curr. Rev. Musculoskelet. Med..

[cit43] Frassica M. T., Grunlan M. A. (2020). ACS Biomater. Sci. Eng..

[cit44] Nie X., Chuah Y. J., Zhu W., He P., Peck Y., Wang D.-A. (2020). Biomaterials.

[cit45] Xue J., He A., Zhu Y., Liu Y., Li D., Yin Z., Zhang W., Liu W., Cao Y., Zhou G. (2018). Biomed. Mater..

[cit46] Nguyen D., Hägg D. A., Forsman A., Ekholm J., Nimkingratana P., Brantsing C., Kalogeropoulos T., Zaunz S., Concaro S., Brittberg M., Lindahl A., Gatenholm P., Enejder A., Simonsson S. (2017). Sci. Rep..

[cit47] Fontana A. (2012). Arthrosc. Tech..

[cit48] Dutta R. C., Dutta A. K. (2009). Biotechnol. Adv..

[cit49] Kleinman H. K., Philp D., Hoffman M. P. (2003). Curr. Opin. Biotechnol.

[cit50] Lutolf M. P., Hubbell J. A. (2005). Nat. Biotechnol..

[cit51] Brandl F., Sommer F., Goepferich A. (2007). Biomaterials.

[cit52] Pennesi G., Scaglione S., Giannoni P., Quarto R. (2011). Curr. Pharm. Biotechnol..

[cit53] Stevens M. M. (2008). Mater. Today.

[cit54] Bergschmidt P., Bader R., Ganzer D., Hauzeur C., Lohmann C. H., Krüger A., Rüther W., Tigani D., Rani N., Esteve J. L., Prats F. L., Zorzi C., Madonna V., Rigotti S., Benazzo F., Rossi S. M. P., Mittelmeier W. (2015). Knee.

[cit55] Meier E., Gelse K., Trieb K., Pachowsky M., Hennig F. F., Mauerer A. (2016). J. Orthop. Surgery Res..

[cit56] Nathwani D., McNicholas M., Hart A., Miles J., Bobić V. (2017). JB JS Open Access.

[cit57] Cowie R. M., Briscoe A., Fisher J., Jennings L. M. (2016). Proc. Inst. Mech. Eng., Part H.

[cit58] Affatato S., Ruggiero A., Merola M., Logozzo S. (2017). Composites, Part B.

[cit59] Eichhorn S., Steinhauser E., Gradinger R., Burgkart R. (2012). Med. Eng. Phys..

[cit60] Ferguson S. J., Visser J. M. A., Polikeit A. (2006). Eur. Spine J..

[cit61] Niinomi M. (1998). Mater. Sci. Eng., A.

[cit62] Turell M. B., Bellare A. (2004). Biomaterials.

[cit63] Camp C. L., Stuart M. J., Krych A. J. (2014). Sports Health.

[cit64] Graham B. T., Moore A. C., Burris D. L., Price C. (2018). J. Biomech..

[cit65] Kraemer J., Kolditz D., Gowin R. (1985). Spine.

[cit66] Moore A. C., Burris D. L. (2017). Osteoarthr. Cartil..

[cit67] Demott C. J., Grunlan M. A. (2022). J. Mater. Chem. B.

[cit68] Gacasan E. G., Sehnert R. M., Ehrhardt D. A., Grunlan M. A. (2017). Macromol. Mater. Eng..

[cit69] Sarabia-Vallejos M. A., Cerda-Iglesias F. E., Terraza C. A., Cohn-Inostroza N. A., Utrera A., Estrada M., Rodríguez-Hernández J., González-Henríquez C. M. (2023). Mater. Des..

[cit70] Means A. K., Dong P., Clubb F. J., Friedemann M. C., Colvin L. E., Shrode C. A., Coté G. L., Grunlan M. A. (2019). J. Mater. Sci.: Mater. Med..

[cit71] Hou Y., Schoener C. A., Regan K. R., Munoz-Pinto D., Hahn M. S., Grunlan M. A. (2010). Biomacromolecules.

[cit72] Gong J. P., Katsuyama Y., Kurokawa T., Osada Y. (2003). Adv. Mater..

[cit73] Gong J. P. (2010). Soft Matter.

[cit74] Haque M. A., Kurokawa T., Gong J. P. (2012). Polymer.

[cit75] Means A. K., Shrode C. S., Whitney L. V., Ehrhardt D. A., Grunlan M. A. (2019). Biomacromolecules.

[cit76] Bonyadi S. Z., Demott C. J., Grunlan M. A., Dunn A. C. (2021). J. Mech. Behav. Biomed. Mater..

[cit77] Demott C. J., Jones M. R., Chesney C. D., Yeisley D. J., Culibrk R. A., Hahn M. S., Grunlan M. A. (2022). Macromol. Biosci..

[cit78] Ali N., Demott C. J., Dingus O. F., Grunlan M. A., Dunn A. C. (2024). Soft Matter.

[cit79] Liu G., Ding Z., Yuan Q., Xie H., Gu Z. (2018). Front. Chem..

[cit80] Nguyen L. H., Kudva A. K., Saxena N. S., Roy K. (2011). Biomaterials.

[cit81] Bovone G., Dudaryeva O. Y., Marco-Dufort B., Tibbitt M. W. (2021). ACS Biomater. Sci. Eng..

[cit82] Roy C. K., Guo H. L., Sun T. L., Ihsan A. B., Kurokawa T., Takahata M., Nonoyama T., Nakajima T., Gong J. P. (2015). Adv. Mater..

[cit83] Frauenlob M., Guo H., Kurokawa T., Gong J. P. (2023). ACS Macro Lett..

[cit84] Milner P. E., Parkes M., Puetzer J. L., Chapman R., Stevens M. M., Cann P., Jeffers J. R. T. (2018). Acta Biomater..

[cit85] Kaneko D., Tada T., Kurokawa T., Gong J. P., Osada Y. (2005). Adv. Mater..

[cit86] Demott C. J., Jones M. R., Chesney C. D., Grunlan M. A. (2023). ACS Biomater. Sci. Eng..

[cit87] ASTM International, Standard Test Methods for Tensile Properties of Plastics by Use of Microtensile Specimens, 2018, **ASTM D1708-18**

[cit88] Li X., Gong J. P. (2024). Nat. Rev. Mater..

[cit89] Zevenbergen L., Gsell W., Cai L., Chan D. D., Famaey N., Vander Sloten J., Himmelreich U., Neu C. P., Jonkers I. (2018). Osteoarthr. Cartil..

[cit90] Jamison D. I. V., Cannella M., Pierce E. C., Marcolongo M. S. (2013). J. Biomech. Eng..

[cit91] Temple D. K., Cederlund A. A., Lawless B. M., Aspden R. M., Espino D. M. (2016). BMC Musculoskeletal Disord..

[cit92] Grodzinsky A. J., Levenston M. E., Jin M., Frank E. H. (2000). Annu. Rev. Biomed. Eng..

[cit93] Yodmuang S., Guo H., Brial C., Warren R. F., Torzilli P. A., Chen T., Maher S. A. (2019). J. Orthop. Res..

[cit94] Martinez-Carranza N., Berg H. E., Hultenby K., Nurmi-Sandh H., Ryd L., Lagerstedt A.-S. (2013). Osteoarthr. Cartil..

