# Supplementary material for: Architecting a partial thickness cartilage substitute with mimetic, self-assembling hydrogels
Source: J Mater Chem B. 2025 Apr 11;13(19):5613–23. doi: 10.1039/d5tb00050e (PMC12013596; doi:10.1039/d5tb00050e)
Supplement: TB-013-D5TB00050E-s001 [file TB-013-D5TB00050E-s001.pdf]

Supplementary Information (SI) for Journal of Materials Chemistry B.
This journal is © The Royal Society of Chemistry 2025

Supporting Information

Architecting a Partial Thickness Cartilage Substitute with Self-assembling Hydrogels

Olivia F. Dingus,[a] Kathleen A. Parrish,[a] Andrew P. Haney,[b] Cesar A. Ramirez,[a] and Melissa A. Grunlan*[a, b, c]

[a.] Department of Biomedical Engineering, Texas A&M University, College Station, TX 77843-3003 (USA)

[b.] Department of Materials Science & Engineering, Texas A&M University, College Station, TX 77843-3003 (USA)

[c.] Department of Chemistry, Texas A&M University, College Station, TX 77843-3003 (USA)

*Corresponding author email: mgrunlan@tamu.edu

Table S1. Triple network (TN) hydrogel compositions

Hydrogel Notation	Composition					
	1st Network [A]	*2nd Network [B]*		*3rd Network [C]*		
	**AMPS**	**NIPAAm**	**AAm (w.r.t. NIPAAm weight)**	**APTAC**	**AMPS**	**BIS (w.r.t. total monomer concentration)**
TN-APTAC				2.0 M		0.10 mol%
90:10				1.8 M	0.2 M	0.10 mol%
70:30				1.4 M	0.6 M	0.10 mol%
50:50	1.5 M	2.0 M	10 wt%	1.0 M	1.0 M	0.10 mol%
30:70				0.6 M	1.4 M	0.10 mol%
10:90				0.2 M	1.8 M	0.10 mol%
TN-AMPS					2.0 M	0.10 mol%
TN-APTAC				2.0 M		0.05 mol%
90:10				1.8 M	0.2 M	0.05 mol%
70:30				1.4 M	0.6 M	0.05 mol%
50:50	1.5 M	2.0 M	10 wt%	1.0 M	1.0 M	0.05 mol%
30:70				0.6 M	1.4 M	0.05 mol%
10:90				0.2 M	1.8 M	0.05 mol%
TN-AMPS					2.0 M	0.05 mol%

(A): 4 mol% BIS crosslinker w.r.t. AMPS concentration (1.5 M), and 0.1 mol% 2-oxo photo-initiator w.r.t. AMPS concentration (1.5 M)

(B): 0.1 mol% BIS crosslinker w.r.t. NIPAAm concentration (2.0 M), and 0.1 mol% 2-oxo photo-initiator w.r.t. NIPAAm concentration (2.0 M)

(C): 0.1 mol% 2-oxo photo-initiator w.r.t. total monomer concentration (2.0 M)

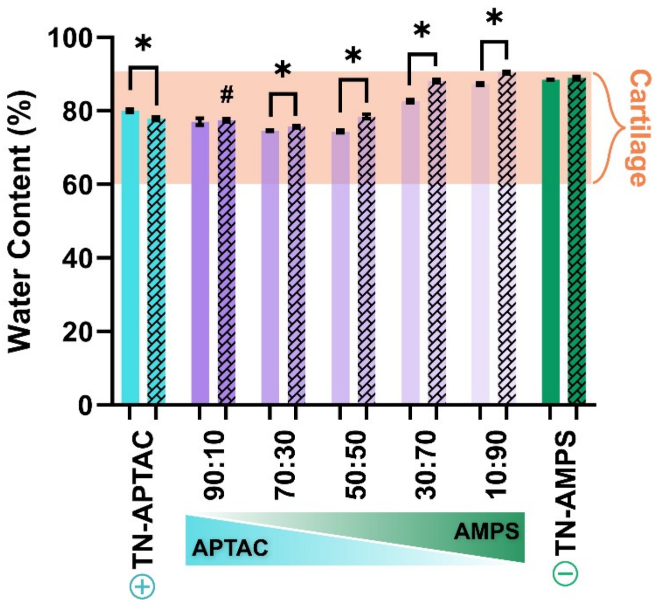

Figure S1. TN hydrogel water content, where solid bars represent TNs with a 3rd network prepared with 0.10 mol% BIS, and dashed bars represent a TNs with a 3rd network prepared with 0.05 mol% BIS. * $p < 0.05$ for TN hydrogels (0.10 mol%) vs. TN hydrogels (0.05 mol% BIS and # $p > 0.05$ for *TN-APTAC* (0.05 mol% BIS) vs. TN hydrogels (0.05 mol% BIS).

Table S2. TN hydrogel equilibrium water content (per **Figure S1**).

Composition	Water Content (%)
0.10 mol% BIS in 3rd network	
TN-APTAC	80.01 ± 0.48
90:10	77.08 ± 0.91
70:30	74.62 ± 0.19
50:50	74.41 ± 0.36
30:70	82.63 ± 0.43
10:90	87.31 ± 0.31
TN-AMPS	88.45 ± 0.07
0.05 mol% BIS in 3rd network	
TN-APTAC	77.81 ± 0.46
90:10	77.42 ± 0.36
70:30	75.68 ± 0.30
50:50	78.39 ± 0.63
30:70	88.10 ± 0.48
10:90	90.45 ± 0.37
TN-AMPS	88.92 ± 0.45

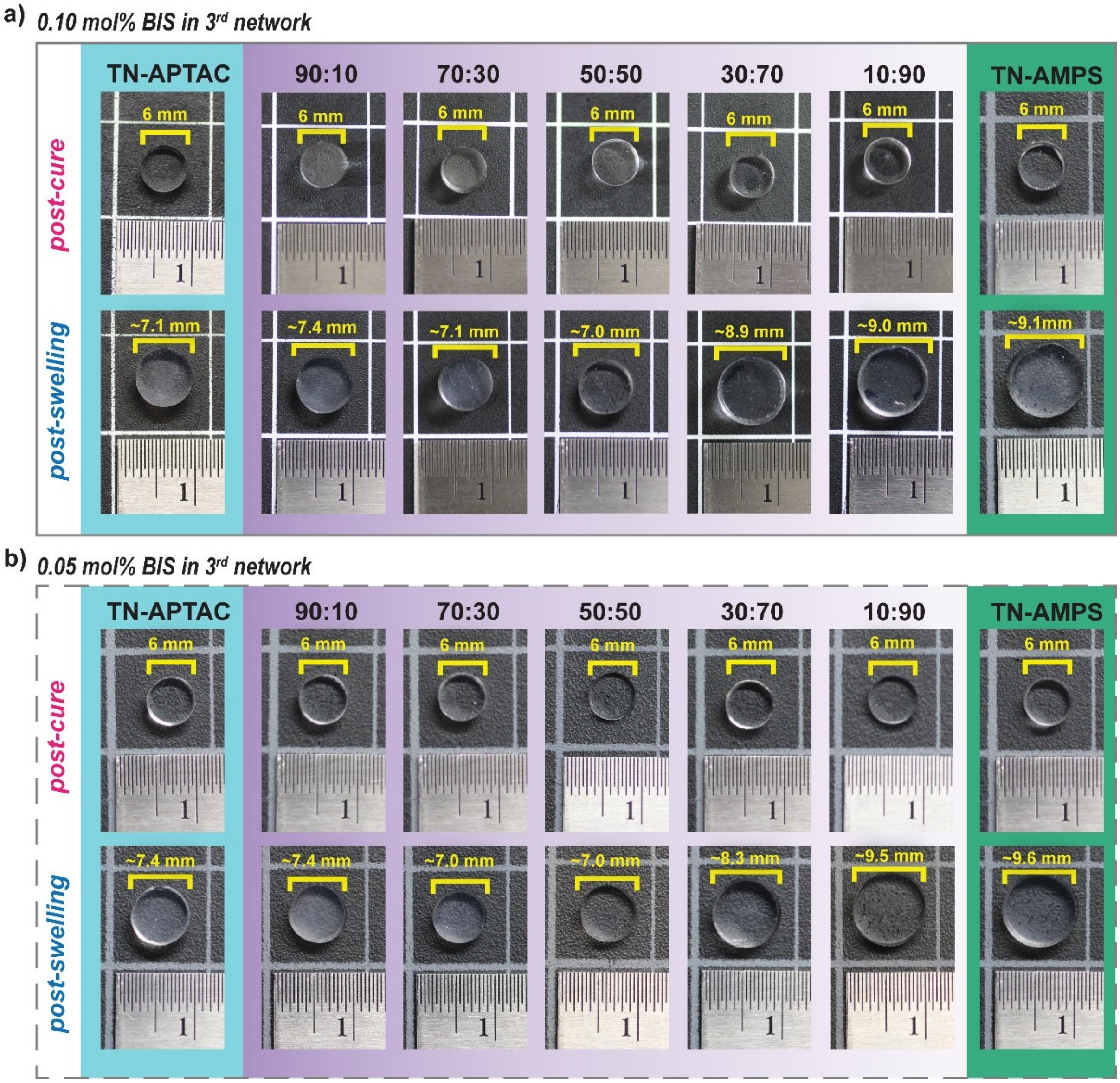

Figure S2. TN hydrogel specimens post-cure (i.e., immediately after curing) and at 7 days post-swelling for: **(a)** 0.10 mol% BIS crosslinker used to form the 3rd network, and **(b)** 0.05 mol% BIS crosslinker used to form the 3rd network.

Table S3. TN hydrogel post-cure mass swelling and diameter increase (per **Figure 3 and Figure S2**).

Composition	Mass Swelling (%)	Diameter Increase (%)
0.10 mol% BIS in 3rd network		
TN-APTAC	82.45 ± 1.97	17.67 ± 2.35
90:10	78.88 ± 2.56	23.45 ± 0.39
70:30	76.36 ± 3.50	18.17 ± 2.91
50:50	54.60 ± 3.54	17.11 ± 0.26
30:70	155.84 ± 0.59	38.45 ± 0.63
10:90	231.01 ± 9.40	49.56 ± 1.23
TN-AMPS	293.86 ± 4.91	60.56 ± 0.82
0.05 mol% BIS in 3rd network		
TN-APTAC	82.54 ± 1.20	22.78 ± 3.54
90:10	77.54 ± 1.70	23.06 ± 2.59
70:30	53.37 ± 0.33	17.00 ± 1.73
50:50	56.80 ± 2.30	15.72 ± 1.84
30:70	185.15 ± 3.40	38.39 ± 0.35
10:90	246.53 ± 9.01	58.28 ± 5.38
TN-AMPS	291.84 ± 8.87	60.56 ± 0.92

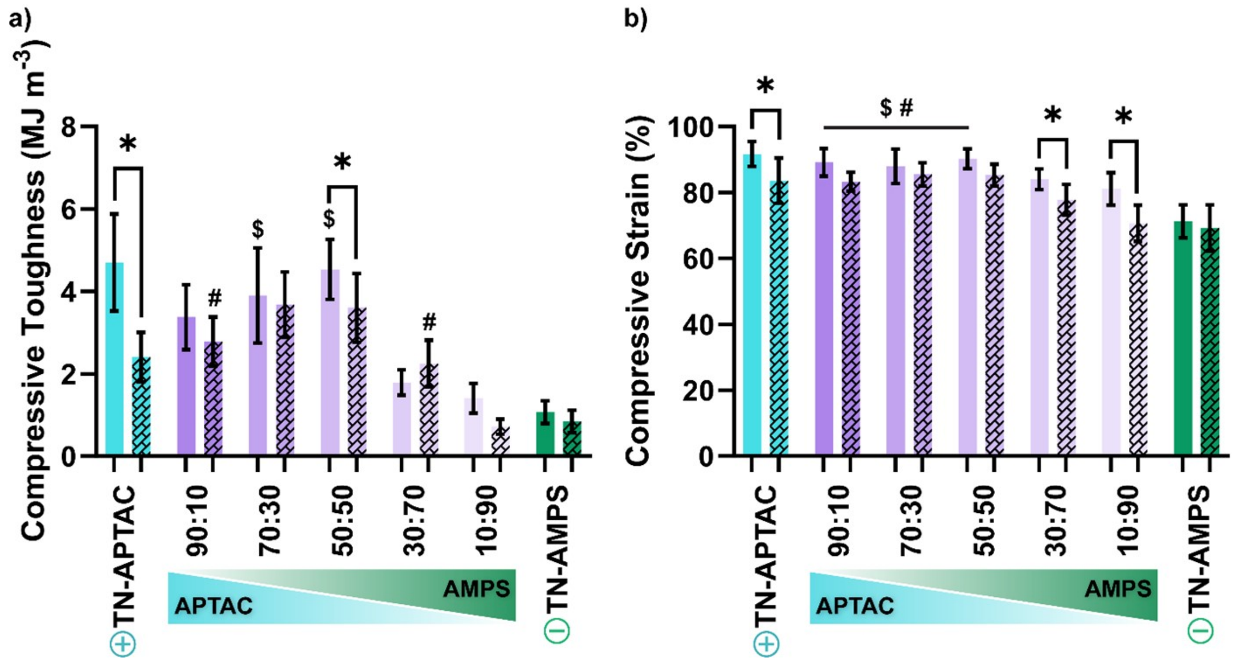

Figure S3. TN hydrogel **(a)** compressive toughness and **(b)** ultimate compressive strain, where solid bars represent TNs with a 3rd network prepared with 0.10 mol% BIS, and dashed bars represent TNs with a 3rd network prepared with 0.05 mol% BIS. ***** $p < 0.05$ for TN hydrogels (0.10 mol% BIS) vs. TN hydrogels (0.05 mol% BIS); **$** $p > 0.05$ for *TN-APTAC* (0.10 mol% BIS) vs. TN hydrogels (0.10 mol% BIS); and **#** $p > 0.05$ for *TN-APTAC* (0.05 mol% BIS) vs. TN hydrogels (0.05 mol% BIS).[1, 2]

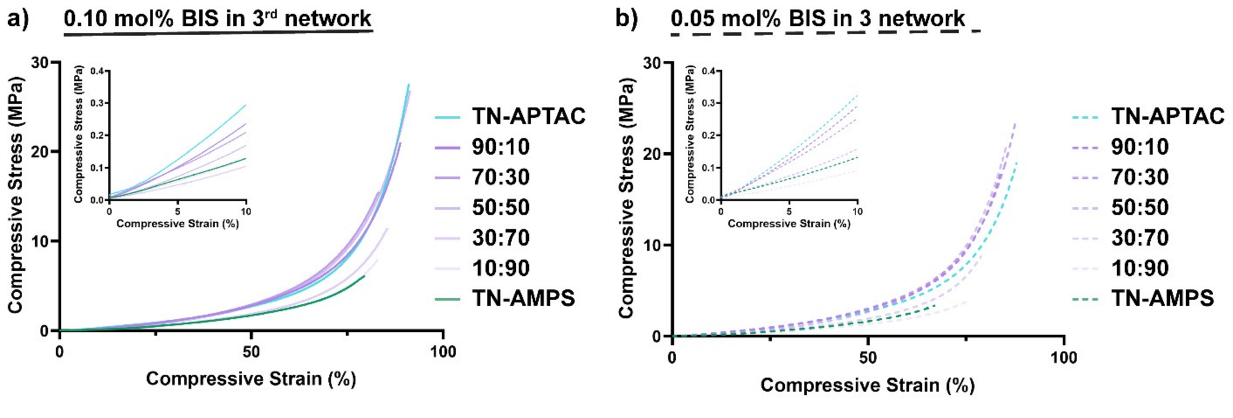

Figure S4. TN hydrogel compressive stress-strain curves of representative samples. Inset graphs highlight the region (0-10% strain) from moduli were calculated as reported in **Figure 4b**.

Table S4. TN hydrogel compressive mechanical properties (per **Figure 4** and **Figure S3**).

**Composition**	**Modulus (MPa) (0-10% ε)**	**Modulus (MPa) (40-50% ε)**	**Modulus (MPa) (70-80% ε)**	**Strength (MPa)**	**Ultimate Strain (%)**	**Toughness (MJ m^{-3})**
0.10 mol% BIS in 3rd network						
TN-APTAC	2.98 ± 0.09	9.55 ± 0.34	52.51 ± 4.88	32.15 ± 10.50	91.69 ± 3.79	4.70 ± 1.18
90:10	2.25 ± 0.27	8.81 ± 0.65	41.09 ± 7.68	20.91 ± 4.78	89.16 ± 4.20	3.38 ± 0.79
70:30	1.83 ± 0.17	10.76 ± 0.39	53.34 ± 3.80	22.76 ± 8.31	87.97 ± 5.23	3.90 ± 1.15
50:50	1.28 ± 0.15	11.21 ± 0.77	63.90 ± 10.17	29.45 ± 5.69	90.23 ± 3.03	4.53 ± 0.73
30:70	1.21 ± 0.16	7.28 ± 0.70	36.93 ± 2.66	13.44 ± 1.93	84.00 ± 3.18	1.79 ± 0.31
10:90	1.25 ± 0.11	5.42 ± 0.34	25.77 ± 2.20	7.13 ± 2.50	81.11 ± 4.92	1.40 ± 0.36
TN-AMPS	1.51 ± 0.04	6.57 ± 0.22	-	5.29 ± 1.79	71.28 ± 5.01	1.07 ± 0.27
0.05 mol% BIS in 3rd network						
TN-APTAC	3.21 ± 0.25	8.68 ± 0.18	45.78 ± 2.40	14.15 ± 7.26	83.64 ± 6.89	2.40 ± 0.61
90:10	2.85 ± 0.14	9.48 ± 0.59	50.28 ± 9.77	15.44 ± 5.21	83.33 ± 2.85	2.78 ± 0.59
70:30	2.40 ± 0.31	11.32 ± 0.40	64.45 ± 6.35	22.56 ± 6.69	85.51 ± 3.53	3.68 ± 0.80
50:50	1.48 ± 0.17	10.85 ± 0.35	72.58 ± 4.32	23.73 ± 6.84	85.32 ± 3.29	3.60 ± 0.83
30:70	1.20 ± 0.08	6.69 ± 0.17	47.25 ± 2.22	8.89 ± 2.65	77.82 ± 4.65	2.25 ± 0.57
10:90	0.86 ± 0.07	4.80 ± 0.28	-	3.40 ± 1.02	70.65 ± 5.49	0.72 ± 0.19
TN-AMPS	1.18 ± 0.09	5.54 ± 0.29	-	4.29 ± 1.72	69.27 ± 7.01	0.84 ± 0.27

Note: Moduli values reported in **Figure 4** calculated from slope of the linear region (0-10% strain) of the stress vs. strain curve (**Figure S4**).

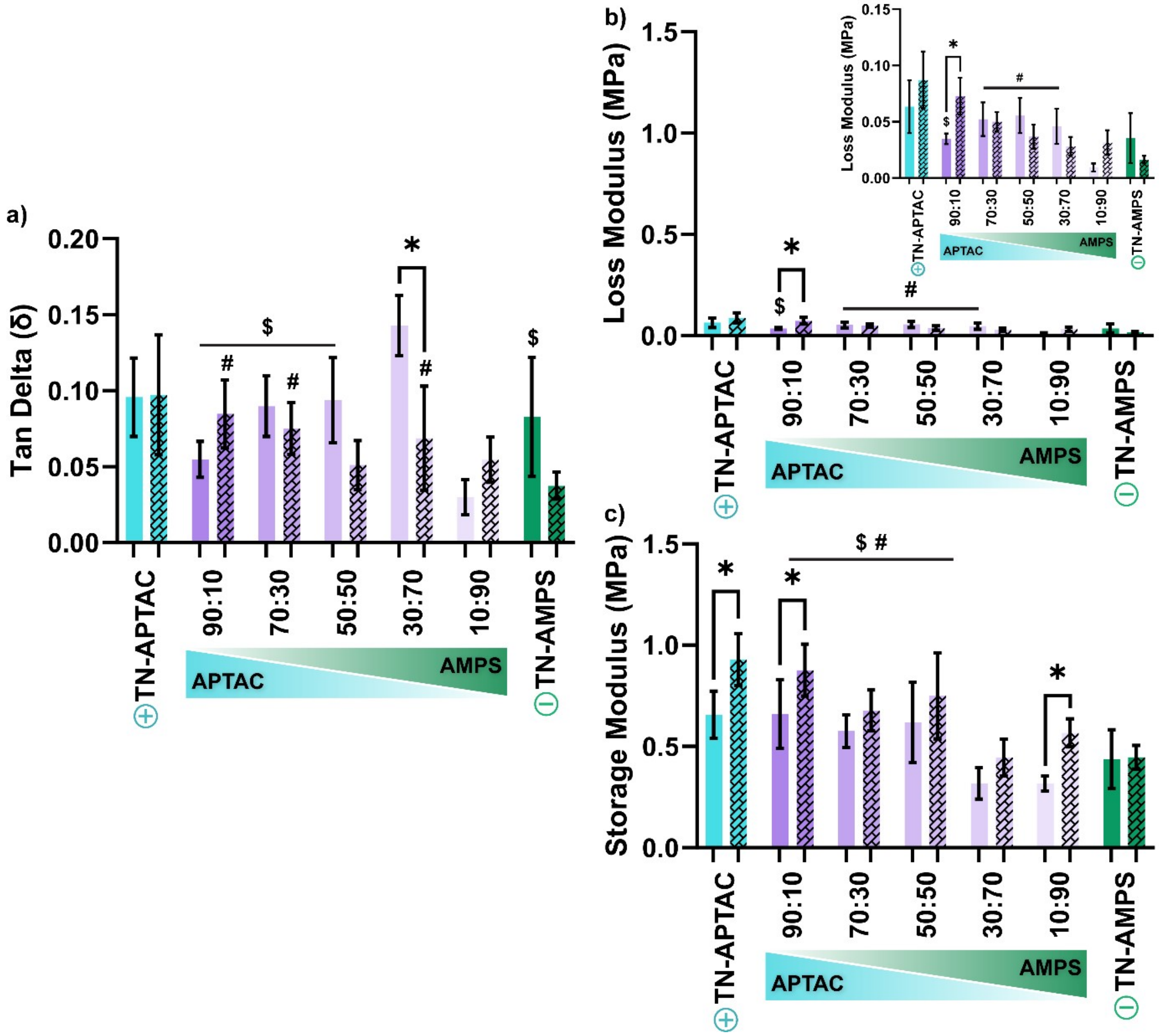

Figure S5. Viscoelastic properties of TN hydrogels, where solid bars represent TNs with a 3rd network prepared with 0.10 mol% BIS, and dashed bars represent TNs with a 3rd network prepared with 0.05 mol% BIS. **(a)** Tan delta, **(b)** loss modulus (where inset graph highlights values from 0 to 0.15 MPa), and **(c)** storage modulus. ***** $p < 0.05$ for TN hydrogels (0.10 mol% BIS) vs. TN hydrogels (0.05 mol% BIS); **$** $p > 0.05$ for *TN-APTAC* (0.10 mol% BIS) vs. TN hydrogels (0.10 mol% BIS); and **#** $p > 0.05$ for *TN-APTAC* (0.05 mol% BIS) vs. TN hydrogels (0.05 mol% BIS).

Table S5. TN hydrogel viscoelastic properties (per **Figure S5**).

Composition	Tan Delta (δ)	Loss Modulus (MPa)	Storage Modulus (MPa)
0.10 mol% BIS in 3rd network			
TN-APTAC	0.096 ± 0.026	0.063 ± 0.023	0.657 ± 0.116
90:10	0.055 ± 0.012	0.035 ± 0.005	0.660 ± 0.170
70:30	0.090 ± 0.020	0.052 ± 0.015	0.576 ± 0.081
50:50	0.094 ± 0.028	0.056 ± 0.016	0.619 ± 0.199
30:70	0.143 ± 0.020	0.046 ± 0.016	0.317 ± 0.078
10:90	0.030 ± 0.012	0.009 ± 0.004	0.316 ± 0.036
TN-AMPS	0.083 ± 0.039	0.036 ± 0.022	0.438 ± 0.144
0.05 mol% BIS in 3rd network			
TN-APTAC	0.097 ± 0.039	0.087 ± 0.025	0.929 ± 0.128
90:10	0.085 ± 0.022	0.073 ± 0.016	0.877 ± 0.129
70:30	0.075 ± 0.017	0.050 ± 0.009	0.678 ± 0.101
50:50	0.051 ± 0.016	0.037 ± 0.011	0.750 ± 0.212
30:70	0.069 ± 0.034	0.028 ± 0.008	0.445 ± 0.092
10:90	0.055 ± 0.015	0.032 ± 0.011	0.567 ± 0.070
TN-AMPS	0.037 ± 0.009	0.016 ± 0.003	0.446 ± 0.059

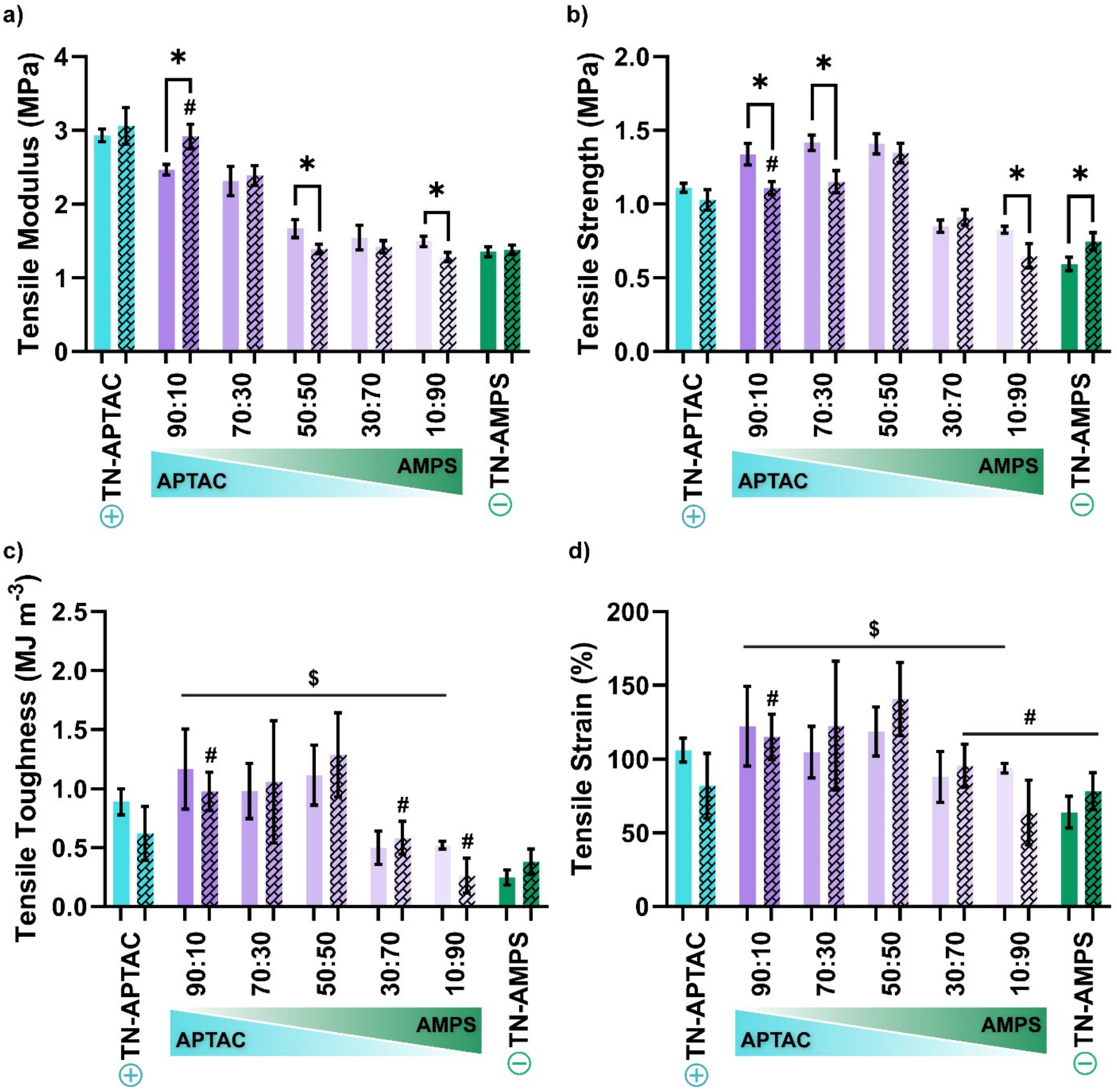

Figure S6. TN hydrogel tensile properties, where solid bars represent TNs with a 3rd network prepared with 0.10 mol% BIS, and dashed bars represent TNs with a 3rd network prepared with 0.05 mol% BIS. **(a)** Tensile modulus, **(b)** tensile strength, **(c)** tensile toughness, and **(d)** ultimate tensile strain. ***** $p < 0.05$ for 0.10 mol% vs. 0.05 mol% BIS TN hydrogels; **$** $p > 0.05$ *TN-APTAC* (0.10 mol% BIS) vs. TN hydrogels (0.10 mol% BIS); **#** $p > 0.05$ *TN-APTAC* (0.05 mol% BIS) vs. TN hydrogels (0.05 mol% BIS).[1, 2]

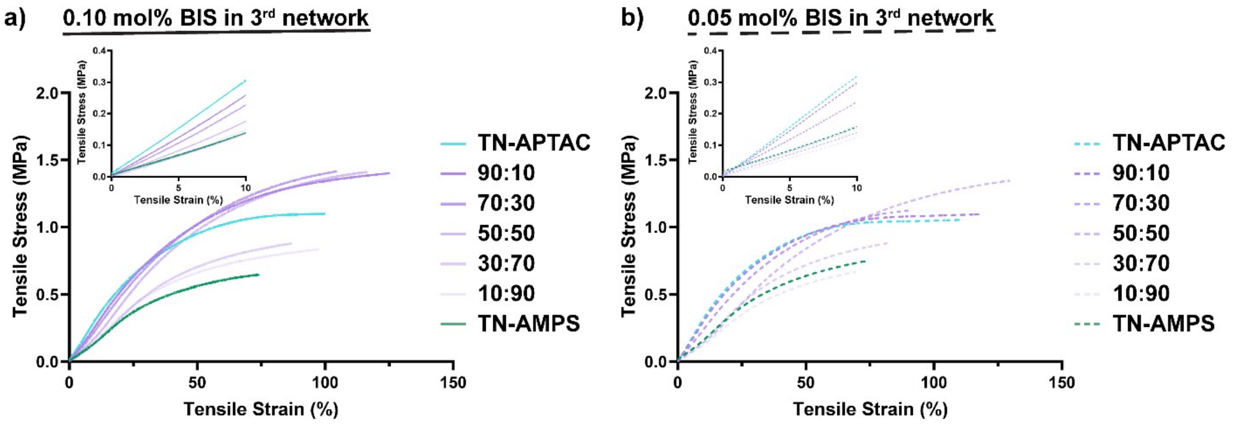

Figure S7. TN hydrogel tensile stress-strain curves of representative samples. Inset graphs highlight the region in which elastic moduli were calculated TN-PA hydrogel tensile stress-strain curves of representative samples. Inset graphs highlight region in which elastic moduli were calculated (per **Figure S6**).

Table S6. TN hydrogel tensile properties (per **Figure S6**).

Composition	Tensile Modulus (MPa)	Tensile Strength (MPa)	Ultimate Strain (%)	Toughness (MJ m^{-3})
0.10 mol% BIS in 3rd network				
TN-APTAC	2.93 ± 0.09	1.11 ± 0.03	106.01 ± 8.05	0.89 ± 0.11
90:10	2.47 ± 0.07	1.34 ± 0.07	122.24 ± 27.00	1.17 ± 0.34
70:30	2.32 ± 0.20	1.42 ± 0.05	104.71 ± 17.48	0.98 ± 0.24
50:50	1.67 ± 0.12	1.41 ± 0.07	118.78 ± 16.61	1.11 ± 0.26
30:70	1.54 ± 0.17	0.85 ± 0.04	87.84 ± 17.31	0.50 ± 0.14
10:90	1.50 ± 0.07	0.83 ± 0.02	93.66 ± 3.23	0.52 ± 0.03
TN-AMPS	1.35 ± 0.07	0.59 ± 0.05	63.98 ± 10.84	0.25 ± 0.06
0.05 mol% BIS in 3rd network				
TN-APTAC	3.06 ± 0.25	1.03 ± 0.07	81.91 ± 21.99	0.62 ± 0.23
90:10	2.92 ± 0.17	1.11 ± 0.04	115.03 ± 15.44	0.98 ± 0.16
70:30	2.39 ± 0.13	1.15 ± 0.08	122.61 ± 43.62	1.06 ± 0.52
50:50	1.39 ± 0.06	1.35 ± 0.07	140.66 ± 24.72	1.29 ± 0.36
30:70	1.42 ± 0.08	0.91 ± 0.05	95.45 ± 14.76	0.58 ± 0.14
10:90	1.28 ± 0.06	0.65 ± 0.08	63.68 ± 22.02	0.26 ± 0.15
TN-AMPS	1.38 ± 0.07	0.75 ± 0.06	78.23 ± 12.51	0.38 ± 0.11

Note: Tensile moduli calculated from slope of the linear region (0-10% strain) of the stress vs. strain curve (**Figure S7**).

Table S7. Qualitative adhesion results for TN hydrogels (0.10 and 0.05 mol% BIS in 3rd network) determined in terms of response of the connection when the construct was orientated by hand vertically (i.e., along a tensile axis): *(i)* no adhesion [***N***] (i.e., no adherence by the connection), *(ii)* slight adhesion [***G***] (i.e., the connection could only withstand gravity), *(iii)* adhesive failure [***A***] (i.e., when tension applied by hand, the connection fails), and *(iv)* cohesive failure [***C***] (i.e., when tension applied by hand, weaker hydrogel fails prior connection). In this way, adhesivity of the connection increased as follows: ***N*** < ***G*** < ***A*** < ***C***. Compositions achieving transitional-like or superficial-like mechanical properties are indicated with "***Transitional**" or "***Superficial**", respectively. ***Circles*** indicate the 3 TN hydrogel pairs (i.e., constructs) subsequently subjected to quantitative lap shear testing.

0.10 mol% BIS in 3rd network

**Compositions**	*TN-APTAC* *Transitional	*90:10* *Transitional	*70:30*	*50:50*	*30:70* *Superficial	*10:90*	*TN-AMPS*
TN-APTAC *Transitional	N	G	G	G	Ⓒ	C	C
90:10 *Transitional	G	N	G	G	Ⓒ	C	C
70:30	G	G	N	G	C	C	C
50:50	G	G	G	N	G	G	G
30:70 *Superficial	Ⓒ	Ⓒ	C	G	N	G	G
10:90	C	C	C	G	G	N	G
TN-AMPS	C	C	C	G	G	G	N

0.05 mol% BIS in 3rd network

**Compositions**	*TN-APTAC*	*90:10*	*70:30* *Transitional	*50:50*	*30:70*	*10:90*	*TN-AMPS*
TN-APTAC	N	G	G	G/A	C	C	C
90:10	G	N	G	A	C	C	C
70:30 *Transitional	G	G	N	G	C	C	C
50:50	G/A	A	G	N	G	G	G
30:70	C	C	C	G	N	G	G
10:90	C	C	C	G	G	N	G
TN-AMPS	C	C	C	G	G	G	N

<table>
<tr><th colspan="5">Three TN Hydrogel Pairs as Candidates for PTCD Bilayered Constructs</th></tr>
<tr><td colspan="2" rowspan="3">Compositions</td><td colspan="3">*Transitional Mimetic</td></tr>
<tr><td colspan="2">0.10 mol% BIS in 3rd network</td><td>0.05 mol% BIS in 3rd network</td></tr>
<tr><td>TN-APTAC</td><td>90:10</td><td>70:30</td></tr>
<tr><td>*Superficial Mimetic</td><td>30:70 (0.10 mol% BIS in 3rd)</td><td>Ⓒ</td><td>Ⓒ</td><td>Ⓒ</td></tr>
</table>

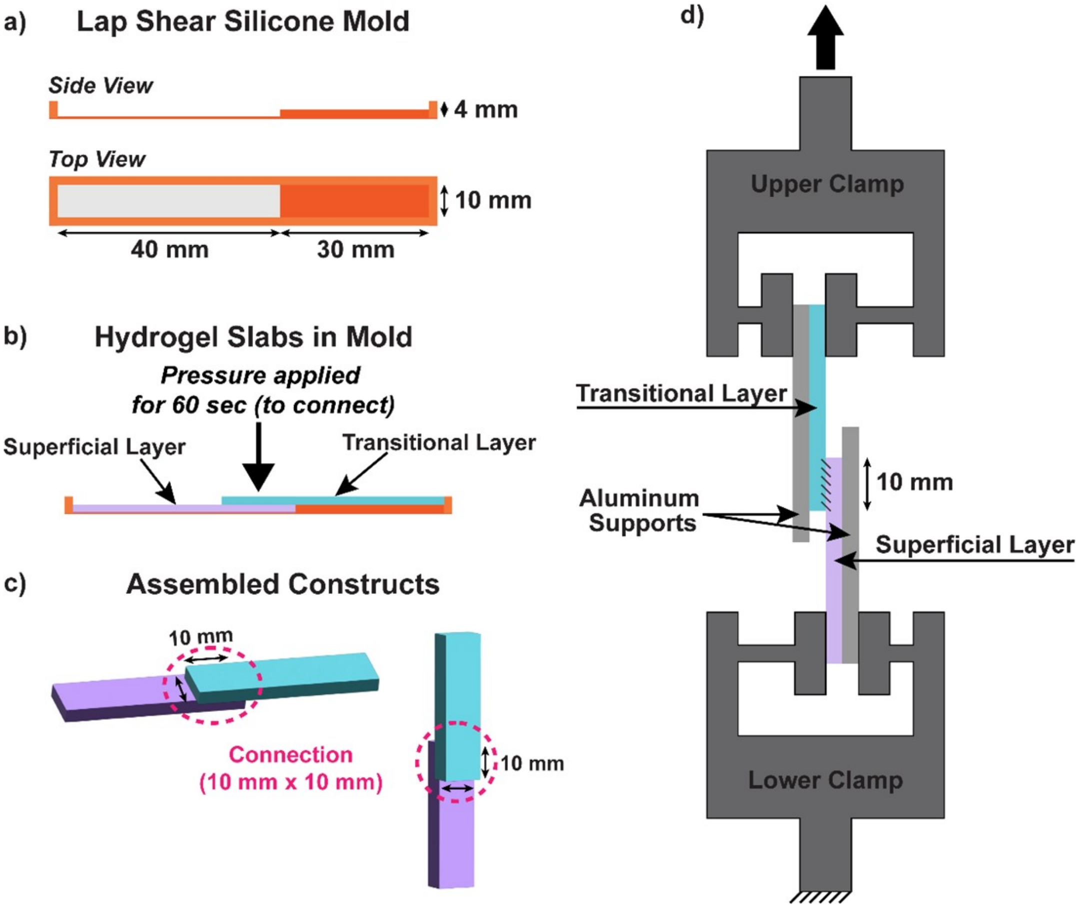

Figure S8. Lap shear strength testing of TN hydrogel constructs: **(a)** silicone mold utilized to ensure a 1 cm^2 overlap (i.e., connection), **(b)** rectangular hydrogel slabs aligned within mold, pressure applied for 60 sec to form connection, **(c)** top and side views of assembled construct, and **(d)** the construct secured within tensile clamps, and vertical alignment provided by aluminum supports; strain applied via movement of lower clamp.

Table S8. TN hydrogel lab shear strength (per **Figure 5**).

Interface	Interfacial Shear Strength (kPa)
TN-APTAC (0.10 mol% BIS) w/ ***30:70*** (0.10 mol% BIS)	101.51 ± 2.34
90:10 (0.10 mol% BIS) w/ ***30:70*** (0.10 mol% BIS)*)*	105.24 ± 14.93
70:30 (0.05 mol% BIS) w/ ***30:70*** (0.10 mol% BIS)	100.63 ± 5.27

References for Support Information:

1. C. J. Demott, M. R. Jones, C. D. Chesney, D. J. Yeisley, R. A. Culibrk, M. S. Hahn and M. A. Grunlan, *Macromol. Biosci.*, 2022, **22**, 2200283.
2. C. J. Demott, M. R. Jones, C. D. Chesney and M. A. Grunlan, *ACS Biomater. Sci. Eng.*, 2023, **9**, 1952-1960.
